# *trans*-IV restriction: a new configuration for metal bis-cyclam complexes as potent CXCR4 inhibitors

**DOI:** 10.1039/d3dt01729j

**Published:** 2024-02-27

**Authors:** Seraj O. Alzahrani, Graeme McRobbie, Abid Khan, Thomas D'huys, Tom Van Loy, Ashlie N. Walker, Isaline Renard, Timothy J. Hubin, Dominique Schols, Benjamin P. Burke, Stephen J. Archibald

**Affiliations:** a Centre for Biomedicine and Positron Emission Tomography Research Centre, Hull York Medical School and University of Hull Cottingham Road Hull HU6 7RX UK stephen.archibald@kcl.ac.uk; b The University of Manchester, Division of Pharmacy and Optometry, School of Health Sciences, Faculty of Biology, Medicine and Health Manchester UK; c KU Leuven, Department of Microbiology, Immunology and Transplantation, Rega Institute, Laboratory of Virology and Chemotherapy Leuven Belgium; d School of Biomedical Engineering and Imaging Sciences, King's College London, 4th Floor Lambeth Wing, St Thomas’ Hospital London SE1 7EH UK; e Department of Chemistry and Physics, Southwestern Oklahoma State University Weatherford OK 73096 USA

## Abstract

The chemokine receptor CXCR4 is implicated in multiple diseases including inflammatory disorders, cancer growth and metastasis, and HIV/AIDS. CXCR4 targeting has been evaluated in treating cancer metastasis and therapy resistance. Cyclam derivatives, most notably AMD3100 (Plerixafor™), are a common motif in small molecule CXCR4 antagonists. However, AMD3100 has not been shown to be effective in cancer treatment as an individual agent. Configurational restriction and transition metal complex formation increases receptor binding affinity and residence time. In the present study, we have synthesized novel *trans*-IV locked cyclam-based CXCR4 inhibitors, a previously unexploited configuration, and demonstrated their higher affinity for CXCR4 binding and CXCL12-mediated signaling inhibition compared to AMD3100. These results pave the way for even more potent CXCR4 inhibitors that may provide significant efficacy in cancer therapy.

## Abstract 2

Alkylation of secondary amines on side-bridged cyclam derivatives locks the resulting metal complex in the *trans*-IV configuration. These derivatives offer new perspectives for the design and development of CXCR4-targeted therapeutic agents.

## Graphical abstract

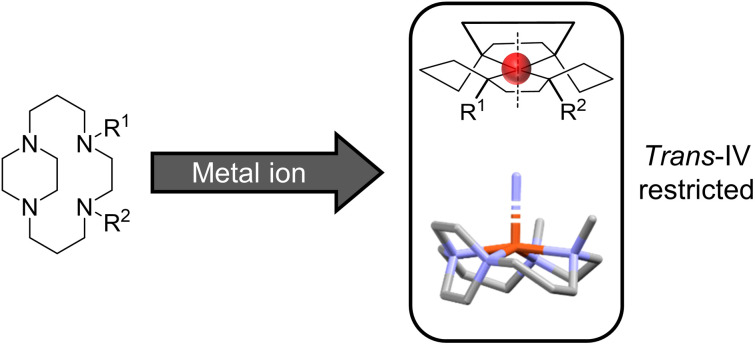

## Introduction

Chemokine C–X–C motif receptor 4 (CXCR4) is a seven transmembrane helix G protein-coupled receptor which is, by interaction with its natural ligand CXCL12 (SDF-1α), essential for both organism development and normal physiological function.^1^ CXCR4 has been implicated in many diseases, including stroke, myocardial infarction and HIV infection.^2–4^ CXCR4 is overexpressed in multiple cancers; promoting tumour growth, metastatic spread to organs with high concentrations of CXCL12 (*e.g.* lung and liver), and resistance to standard chemo/radio/immuno-therapy.^5–8^ This results in particular relevance for clinical molecular imaging of cancers.^9–12^

**Fig. 1:**
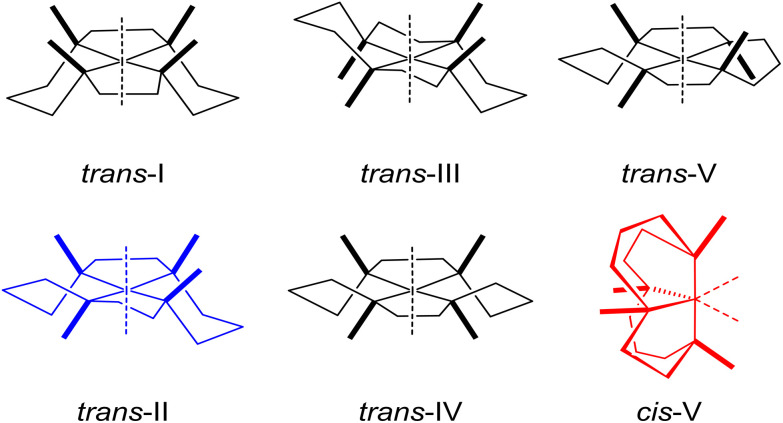
Six possible configurations of 1^st^ row transition metal cyclam complexes.

There is current interest in high affinity antagonists which bind to the CXCR4 receptor to prevent HIV entry and/or abrogate CXCL12 signalling.^13^ Preventing CXCL12-initiated CXCR4 signalling has resulted in a reduction in tumour growth, metastasis inhibition and increase in sensitivity to standard therapies in breast cancer, pancreatic cancer and gliomas.^14–16^ AMD3100 (Plerixafor™) is a cyclam-based CXCR4 antagonist which is used clinically, in combination with G-CSF, as a hematopoietic stem cell mobilization agent. However, AMD3100 lacks efficacy in cancer treatment and more potent CXCR4 inhibitors may lead to more effective receptor-mediated cancer therapy.^13^ The cyclam macrocycles in AMD3100 have the ideal cavity size for complex formation with transition metals, and some AMD3100 transition metal complexes have increased CXCR4 binding affinity in comparison to the metal-free compounds.^17^ Upon metal ion binding, flexible cyclam units can have six possible configurations in solution (Fig. 1), with *trans*-III the most stable due to its lower strain energy,^18^ leading to a stochastic overall affinity reflective of the equilibrium between configurations. In addition, transition metal cyclam complexes exhibit low kinetic and thermodynamic stabilities, precluding their use *in vivo*.^19,20^

Configurational restriction, to lock tetraazamacrocycle derivatives into one configuration rather than being in equilibrium, can be used to optimize amino acid side-chain based receptor coordinate bond interactions and is a route to formation of high affinity antagonists with longer receptor residence times and increased metal complex stabilities.^21–28^

**Fig. 2:**
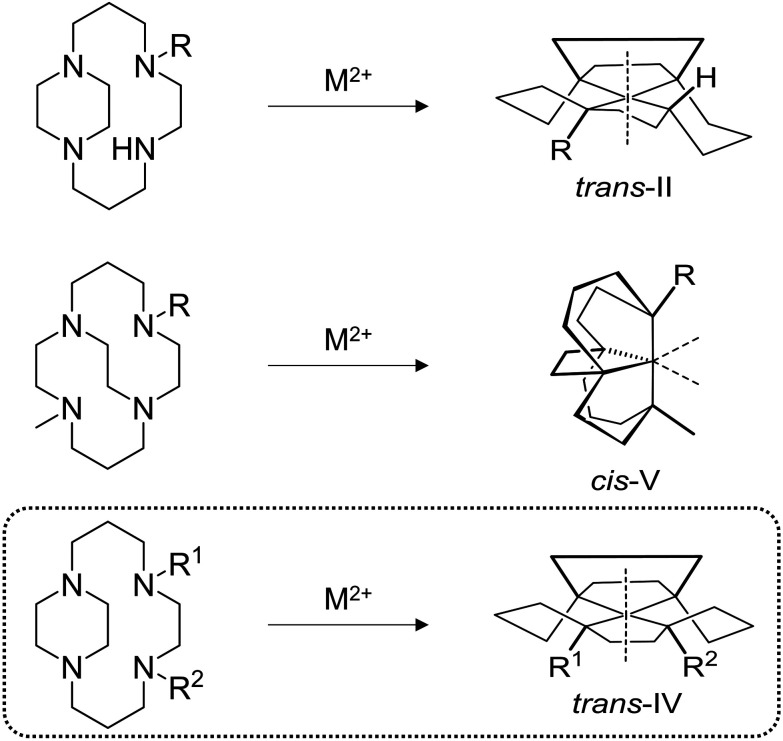
*N*-Functionalization patterns of the side-bridged and cross-bridged cyclam macrocycles and their corresponding configurations for 1^st^ row transition metal complexes. R = alkyl, R^1^, R^2^ ≠ H; M = Cu, Ni, Zn.

Linking adjacent cyclam nitrogen atoms with an ethylene bridge to form a piperazine ring can be carried out to form “side-bridged” derivatives, which, upon transition metal complex formation, are locked in the *trans*-II configuration (Fig. 2). Side-bridged (SB) *trans*-II restricted copper(ii) and zinc(ii) complexes demonstrate superior CXCR4-mediated anti-HIV properties compared with their respective AMD3100 analogues.^21–25^ Linking of non-adjacent nitrogen atoms with an ethylene linker to form “cross-bridged” (CB) derivatives, and transition metal complex formation, provides macrocycles which are exclusively locked in the *cis*-V configuration (Fig. 2). The copper(ii) complexes of CB-Bicyclam have multiple advantages over AMD3100 and Cu_2_AMD3100, including increased affinity, longer receptor residence times and increased complex stability.^26,29^

In the present work, we demonstrate a route to the formation of a third generation of configurationally restricted CXCR4 antagonists, employing strategies developed by Kaden and co-workers to exclusively form the *trans*-IV configuration (Fig. 2).^30,31^ The general synthetic methodology is presented, followed by three significantly different novel examples to show how this methodology can be applied. Biological evaluation of the novel transition metal complexes was carried out in multiple assays to assess inhibitory properties of molecules with this configuration against the CXCR4 receptor.

## Results and discussion

### Synthesis of *trans*-IV configurationally restricted compounds

**Scheme 1:**
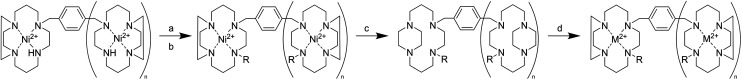
General scheme for the formation of *trans*-IV cyclam complexes; (a) *n*-BuLi, DMSO, (b) MeI, (c) NaCN, MeCN/H_2_O, (d) M(OAc)_2_, MeOH (*n* = 0 or 1).

The formation of novel *trans*-IV derivatives follows a general synthetic pathway (Scheme 1). First, the nickel(ii) *trans*-II complex is alkylated in a two-step *in situ* process. *n*-BuLi is used to deprotonate the secondary amine to form the nickel(ii) stabilized amido complex, which can subsequently be alkylated using an alkyl halide. After formation of the nickel(ii) *trans*-IV species, the free ligand can be synthesized by decomposing the nickel(ii) complex using NaCN, which can subsequently be reacted with transition metal salts to form complexes with other metal centres.

**Fig. 3:**
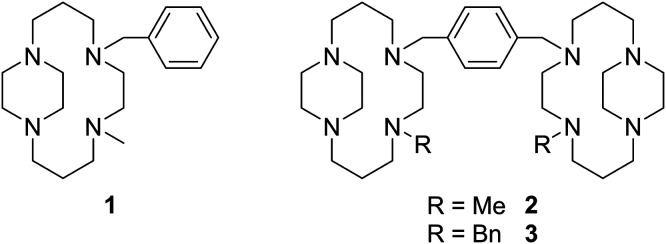
Novel ligands synthesized following the described general synthetic pathway.

To develop and optimise the methodology, the synthesis of a mono-cyclam derivative 1 was first attempted (Fig. 3). The side-bridged NH precursor was synthesized in three steps following our previously described protocols.^25,27,32^ Formation of the *trans*-II nickel(ii) complex was carried out using nickel(ii) nitrate in dry methanol, using methodologies we have described previously.^27^ Alkylation of the secondary amine to form the novel *trans*-IV structures was carried out by a method analogous to that first described by Kaden and co-workers.^30^ The secondary amine nickel(ii) complex was deprotonated using *n*-BuLi in dry DMSO. Successful deprotonation can be clearly observed by noting the colour change from orange of the starting material to blue of the amido derivative, which is stable in aprotic conditions. The amido complex can subsequently be alkylated using methyl iodide, with observation of an orange colour indicating product formation. The complex [Ni1]^2+^ can then be isolated as the hexafluorophosphate salt. The alkylated *trans*-IV nickel(ii) complex can be decomposed to form the free ligand 1 using sodium cyanide, in a method similar to that used for nickel(ii) templated cyclam synthesis,^33^ with minor synthetic modifications introduced due to the lower solubility of the hexafluorophosphate complex. Complex formation reactions with zinc(ii) and copper(ii) acetate were then be carried out following our previously described methodology to synthesise *trans*-II complexes,^21,22,26^ with the *trans*-IV complexes forming in good yields.

**Fig. 4:**
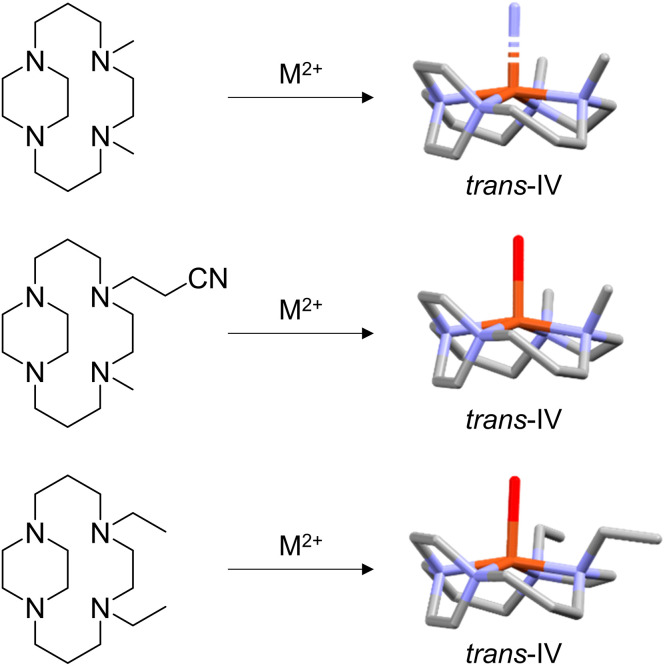
Examples of *N*-functionalization of the side-bridged cyclam resulting in *trans*-IV configuration of the M^2+^ metal complex and associated crystal structures.

In our previous studies we have shown that either mechanical restriction (*cis*-V) or sterics (*trans*-II) can fix the configuration of the cyclam complex on addition of an ethylene bridge between ring nitrogen donor atoms. Examination of previously determined X-ray crystal structures of alkylated side-bridge cyclam complexes demonstrates that the *trans*-IV configuration is adopted, although this was not highlighted at the time of publication. A search of the CCDC found a total of three deposited single crystal X-ray structures of transition metal complexes where a side-bridged cyclam chelator had been alkylated in a similar synthetic procedure to give related compounds. Despite variations in substituents on the cyclam nitrogens (methyl, ethylcyano or ethyl), all three structures clearly adopt the *trans*-IV configuration (Fig. 4). In contrast to the *trans*-II complexes, this steric restriction favours the two non-bridged nitrogen methyl groups oriented on the same side of the macrocyclic ring plane with the side bridge piperazine carbons oriented to the other side of the plane. These complexes offer the opportunity to probe the biological impact of this fixed configuration on coordination interactions with the CXCR4 chemokine receptor and utilising biological assays to determine binding potency and inhibitory activity.

Mono-ring configurationally restricted macrocyclic complexes are not ideal as CXCR4 antagonists as they can interact with only one receptor amino acid side-chain *via* a coordination interaction.^28^ Bis-macrocyclic complexes have significantly higher affinities as the metal ions can form coordination interactions with both Asp^171^ and Asp^262^.^34,35^ The methodologies developed for the synthesis of 1 were successfully translated to the bis-macrocyclic analogue 2 without modification (Fig. 3). The three-step ligand-to-ligand reactions from the secondary amine to the tetra-alkylated amine was carried out in an overall yield of 76% using gram amounts. Identity and purity were confirmed by analysis using ^1^H NMR, mass spectrometry and elemental analysis.

It is of interest to ascertain if the methodology described to synthesize *trans*-IV bis-macrocyclic CXCR4 antagonists is limited to highly reactive alkylating agents such as methyl iodide or has wider utility. To assess this, methyl iodide used in the synthesis of 2 was replaced with benzyl bromide, containing a less reactive halogen leaving group and bulkier substituent, to form 3 (Fig. 3). Reaction conditions did not need to be modified and 3 could be synthesized as easily as 2 in a 69% three-step ligand-to-ligand overall yield. Versatility in substitution indicates the potential for formation of a wider range of derivatives of bis-macrocyclic *trans*-IV CXCR4 antagonists, allowing for variables such as lipophilicity, π–π stacking ability and steric effects to be evaluated for binding effects. Alkylation with a protected functional group may also allow for further functionalization.^24,27,32^

### *In vitro* evaluation of the CXCR4 affinity of the novel *trans*-IV metal complexes

The synthesised *trans*-IV complexes were screened for their CXCR4 binding properties using three *in vitro* assays: anti-HIV replication, CXCL12 competition binding and CXCL12-induced calcium signalling. CXCR4 is one of the two main co-receptors involved in the infection process of HIV-1.^36,37^ The effect of the compounds was tested on the viral infection of the CXCR4-using HIV-1 strain NL4-3 (×4). Binding competition with the natural chemokine (CXCL12) binding was directly determined using AlexaFluor647-labelled CXCL12.^38,39^ As a measure of abrogation of the natural CXCR4/CXCL12 signalling pathway, a functional downstream assay can be carried out to measure Ca^2+^ ions released from the endoplasmic reticulum.^40,41^ All assays were carried out over a concentration range and are presented as IC_50_ values (Table 1).

**Table 1:** CXCR4-dependent effects and cellular toxicity of *trans*-IV metal complexes

	CXCR4-mediated effects, IC_50_ (nM)			CC_50_ (μM)
	Anti-HIV activity^a^	CXCL12 binding^b^x	Ca^2+^ signalling^c^	Cellular toxicity^d^
1	>5000	>1000	>10 000	>100
[Ni1]^2+^	>5000	>3000	>2000	26.0 ± 6.4
[Cu1]^2+^	>5000	>10 000	>2000	68.2 ± 3.4
[Zn1]^2+^	>5000	>1000	>2000	>100
2	>5000	>1000	>10 000	>100
[Ni_2_2]^4+^	>5000	>1000	>2000	11.7 ± 2.8
[Cu_2_2]^4+^	>5000	>1000	>2000	37.9 ± 7.1
[Zn_2_2]^4+^	642.0 ± 193.7	5.5 ± 0.7	67.5 ± 38.9	46.7 ± 4.0
3	>5000	>1000	>10 000	>50
[Ni_2_3]^4+^	>5000	668.0 ± 106.1	>2000	10.6 ± 0.7
[Cu_2_3]^4+^	332.5 ± 6.4	25.5 ± 3.5	108.0 ± 11.3	32.7 ± 0.3
[Zn_2_3]^4+^	327.0 ± 25.5	4.5 ± 0.7	72.5 ± 38.9	40.7 ± 0.2
AMD3100	20.0 ± 1.4	18.0 ± 4.2	203.5 ± 19.4^29^	>100
[Ni_2_AMD3100]^4+^	7.9^42^^,^^e^	ND	2.0^42^^,^^e^	>100^42^^,^^e^
[Cu_2_AMD3100]^4+^	22.4^42^^,^^e^	ND	48.7^42^^,^^e^	>100^42^^,^^e^
[Zn_2_AMD3100]^4+^	6.8^42^^,^^e^	ND	2.9^42^^,^^e^	>100^42^^,^^e^

a Concentration required to inhibit the replication of HIV-1 strain NL4-3 (×4) by 50% in MT-4 cells.

b Concentration required to inhibit CXCL12 binding by 50%.

c Concentration required to reduce the level of Ca^2+^ ions observed during a normal signalling process by 50% in U87.CD4.CXCR4 cells.

d Concentration required to reduce cell viability by 50% in MT-4 cells.

e Concentration values published in μg mL^−1^ converted to nM.

AMD3100 binds to CXCR4 *via* hydrogen bonding to aspartate residues 171 and 262.^34^ Tetra-alkylated macrocyclic ligands have very low hydrogen bonding potential and therefore demonstrate no measurable affinity towards the CXCR4 receptor (Table 1). Mono-macrocyclic transition metal complexes are known to weakly bind to the receptor,^27,28^ relative to our high affinity bis-macrocycles. In this study, mono-macrocyclic complexes [Ni1]^2+^, [Cu1]^2+^ and [Zn1]^2+^ demonstrated micromolar level binding in the CXCL12 binding assay and no measurable activity up to 5 μM or 2 μM for anti-HIV activity and calcium signalling, respectively. Hence, they are characterized as relatively low affinity binding agents. For the methyl substituted bis-macrocyclic *trans*-IV complexes (2), the zinc(ii) complex is the only compound to demonstrate any significant affinity/activity; with the copper(ii) and nickel(ii) complexes showing similar activity to their mono-ring counterparts.

Modification of the methyl substituent to a benzyl (3) on either of the nickel(ii) or zinc(ii) complex does not significantly alter the activity in these assays, however an increase in potency is observed for the copper(ii) complex, giving it a similar profile to that of [Zn_2_3]^4+^. [Zn_2_2]^4+^ and [Zn_2_3]^4+^ are more effective at blocking CXCL12 interactions with the receptor than AMD3100 in both the signalling and ligand binding assays but are less potent in the anti-HIV assay. All compounds were assessed in cellular toxicity assays and have CC_50_ values >10 μM.

Comparison can also be made to the published assay values for the metal complexes of AMD3100 in related assays, showing a similar trend to AMD3100, with higher potency for [M_2_AMD3100]^4+^ in the anti-HIV assay compared to the *trans*-IV complexes. The calcium signalling assay shows that significant disruption of the binding interaction has occurred for the nickel(ii) complex [Ni_2_3]^4+^ relative to [Ni_2_AMD3100]^4+^. Further comparison in the CXCL12 binding assay would be useful to determine any differences in the binding inhibition and residence time assays are needed to better understand the potential *in vivo* properties. The *trans*-IV configuration may confer additional advantages in terms of residence time and complex stability due to the structural rigidity.^26^

## Experimental

### General information

Bulk solvent was removed by evaporation under reduced pressure and trace solvent was removed using a Schlenk line. NMR spectra were recorded with a Jeol ECZ 400S spectrometer (400.2 MHz for ^1^H and 100.6 MHz for ^13^C). Chemical shifts (*δ*) are reported in ppm, using internal TMS or residual non-deuterated solvent signal as standards. High- and low-resolution mass spectra were recorded using an electrospray ion-trap LC-MS in positive mode. Elemental analysis was performed using a CHN analyser EA1108 (Carlo Erba). UV-vis spectra were obtained using an Agilent 800 diode spectrometer. Side-bridged secondary amine ligands were synthesised following our previously reported methods.^21,25,27^

#### Nickel(ii) 5-benzyl-1,5,8,12-tetraazabicyclo[10.2.2]hexadecane complex

5-Benzyl-1,5,8,12-tetraazabicyclo[10.2.2]hexadecane (1.10 g, 3.47 mmol) was dissolved in degassed anhydrous MeOH (25 mL). An anhydrous methanolic (10 mL) solution of nickel(ii) nitrate hexahydrate (1.11 g, 3.82 mmol) was added dropwise and the mixture was refluxed under argon for 12 h. Solvent was removed *in vacuo* and the macrocycle complex was purified *via* size exclusion chromatography (Sephadex LH20) in MeOH to yield bright orange crystals (1.51 g, 87%). Elemental analysis: calculated (%) for [C_19_H_32_N_4_Ni](NO_3_)_2_·0.1H_2_O·0.1MeOH: C, 45.50; H, 6.52; N, 16.67. Found (%): C, 45.51; H, 6.77; N, 16.65. MS (ESI): *m*/*z* = 436.19 ([[C_19_H_32_N_4_Ni](NO_3_)]^+^). HRMS (ESI): calculated for [C_19_H_32_N_4_Ni]^2+^, 187.0985; found 187.0981.

#### Di-nickel(ii) 1,4-bis(1,5,8,12-tetraazabicyclo[10.2.2]hexadecan-5-yl)xylene complex

First reported by Smith *et al.*^27^ Amounts used: 1,4-bis(1,5,8,12-tetraazabicyclo[10.2.2]hexadecan-5-yl)xylene (3.3 g, 5.95 mmol) in dry MeOH (50 mL), an anhydrous methanolic (20 mL) solution of nickel(ii) nitrate hexahydrate (3.80 g, 13.08 mmol). To yield bright orange crystals (4.91 g, 90%). Elemental analysis: calculated (%) for [C_32_H_58_N_8_Ni_2_](NO_3_)_4_·0.5H_2_O·2MeOH: C, 41.11; H, 6.80; N, 16.92. Found: C, 41.07; H, 6.77; N, 16.98. MS (ESI): *m*/*z* = 857.40 ([[C_32_H_58_N_8_Ni_2_](NO_3_)_3_]^+^). HRMS (ESI): calculated for [C_32_H_58_N_8_Ni_2_]^4+^, 167.5867; found 167.5864. UV-vis (H_2_O): *λ*_max_ = 526 nm.

### General procedure for *N*-alkylation of Ni(ii) complex to generate *trans*-IV structures

The nickel(ii) complex was dissolved in dry DMSO (15 mL) and stirred *in vacuo* for 1 h. The orange solution was purged with argon for 10 minutes. A solution of *n*-butyl lithium (2.5 M) in hexane was added dropwise over 30 min. A blue colour developed and the mixture was stirred at room temperature for 30 min. Alkyl halide was added dropwise and an orange solution re-appeared, which was stirred at room temperature for 1 h. The solution was stirred under vacuum again to remove hexane and alkyl halide. The DMSO solution was added dropwise to a saturated solution of ammonium hexafluorophosphate in ethanol. The orange precipitate was filtered and washed with diethyl ether (100 mL). The orange solid was dissolved in acetonitrile and evaporated *in vacuo* to yield an orange crystalline solid.

#### [Ni1]^2+^

Amounts used: Nickel(ii) 5-benzyl-1,5,8,12-tetraaza-bicyclo[10.2.2]hexadecane nitrate (1.2 g, 2.4 mmol), *n*-butyl lithium (2.5 M) in hexane (0.4 mL, 4.8 mmol, 2 equiv.), methyl iodide (1.36 g, 9.6 mmol, 4 equiv.), ammonium hexafluorophosphate (40 equiv.) in ethanol (40 mL). Yield (1.5 g, 92%). Elemental analysis: calculated (%) for [C_20_H_34_N_4_Ni](PF_6_)_2_·2.2H_2_O·0.15 MeCN: C, 33.63; H, 5.40; N, 8.02. Found (%): C, 33.71; H, 5.70; N, 8.01. MS (ESI): *m*/*z* = 533.19 ([[C_20_H_34_N_4_Ni](PF_6_)]^+^). HRMS (ESI): calculated for [[C_20_H_34_N_4_Ni](NO_3_)]^+^, 450.2010; found 450.1998.

#### [Ni_2_2]^4+^

Amounts used: Di-nickel(ii) 1,4-bis(1,5,8,12-tetraazabicyclo[10.2.2]hexadecan-5-yl)xylene complex (1.5 g, 1.63 mmol), *n*-butyl lithium (2.5 M) in hexane (0.6 ml, 6.52 mmol, 4 equiv.), methyl iodide (1.85 g, 13.04 mmol, 8 equiv.), ammonium hexafluorophosphate (60 equiv.) in ethanol (60 mL). Yield (1.88 g, 90%). Elemental analysis: calculated (%) for [C_34_H_62_N_8_Ni_2_](PF_6_)_4_·2.4H_2_O: C, 30.86; H, 5.09; N, 8.47. Found: C, 30.80; H, 5.12; N, 8.61. MS (ESI): *m*/*z* = 991.08 ([[C_34_H_62_N_8_Ni_2_](H)(PF_6_)_2_]^+^). HRMS (ESI): calculated for [[C_34_H_62_N_8_Ni_2_](CH_3_COO)_2_]^2+^, 408.2030; found 408.2028.

#### [Ni_2_3]^4+^

Amounts used: Di-nickel(ii) 1,4-bis(1,5,8,12-tetraazabicyclo[10.2.2]hexadecan-5-yl)xylene complex (1.2 g, 1.30 mmol), *n*-butyl lithium (2.5 M) in hexane (0.5 mL, 0.33 g, 5.22 mmol, 4 equiv.), benzyl bromide (1.78 g, 10.4 mmol, 8 equiv.), ammonium hexafluorophosphate (60 equiv.) in ethanol (60 mL). Yield (1.01 g, 85%). Elemental analysis: calculated (%) for [C_46_H_70_N_8_Ni_2_](PF_6_)_4_·1.4H_2_O: C, 37.91; H, 5.03; N, 7.69. Found: C, 37.88; H, 5.02; N, 7.60. MS (ESI): *m*/*z* = 570.19 ([[C_46_H_70_N_8_Ni_2_](PF_6_)_2_]^2+^). HRMS (ESI): calculated for [[C_34_H_62_N_8_Zn_2_](NO_3_)_2_]^2+^, 487.2088; found 487.2075.

### General procedure for free ligand formation

The nickel(ii) complex was dissolved in a mixture of water/MeCN (90 : 10) to form a bright orange solution. Sodium cyanide was added to the orange solution in one portion, the mixture became less intensely coloured and was heated at 80 °C for 24 h. The resulting mixture was cooled to room temperature, then in an ice bath. The nickel cyanide salt was removed by filtration and the filtrate was concentrated *in vacuo* to remove MeCN. KOH was added to the aqueous residue to adjust the pH to 14. The basic aqueous layer was extracted with DCM (5 × 40 mL). The combined organic layers were dried over MgSO_4_, filtered and concentrated *in vacuo* to yield a colourless oil, which was dried on a Schlenk line overnight to yield a white solid.

#### Ligand 1

Amounts used: [Ni1]^2+^ (1.4 g, 2.06 mmol) in 10 mL water/MeCN, sodium cyanide (400 mg, 8.24 mmol). Yield (622 mg, 91%). ^1^H NMR (CDCl_3_): *δ* 7.45–7.23 (m, 5H, Ar–H), 4.87 (s, 2H, CH_2_–Ar), 4.33 (s, 4H, CH_2_–N), 4.24–4.18 (m, 2H, CH_2_–N), 3.12–3.01 (m, 2H, CH_2_–N), 2.86 (s, 2H, CH_2_–N), 2.70–2.58 (m, 5H, CH_2_–N), 2.43–2.19 (m, 5H, CH_2_–N), 1.81 (s, 3H, CH_3_), 1.54 (m, 2H, CH_2_– –N), 1.48 (m, 2H, CH_2_– –N). ^13^C NMR (CDCl_3_): *δ* 23.51 (N–CH_2_), 25.62 (N–CH_2_), 44.88 (N–CH_3_), 48.19 (N–CH_2_), 50.21 (N–CH_2_), 55.32 (N–CH_2_), 55.71 (N–CH_2_), 56.46 (N–CH_2_), 56.91 (N–CH_2_), 61.11 (CH_2_–Ar), 124.32 (Ar–C), 129.51 (Ar–CH), 129.82 (Ar–CH), 143.22 (Ar–C). Elemental analysis: calculated (%) for C_20_H_34_N_4_·2.3H_2_O: C, 64.58; H, 10.46; N, 15.06. Found: C, 64.20; H, 10.37; N, 15.19. MS (ESI): *m*/*z* = 331.2 ([M + H]^+^). HRMS (ESI): calculated for [M + H]^+^, 331.2856; found 331.2849.

#### Ligand 2

Amounts used: [Ni_2_2]^4+^ (1.8 g, 1.40 mmol) in 20 mL water/MeCN, sodium cyanide (700 mg, 14 mmol). Yield (770 mg, 94%). ^1^H NMR (CDCl_3_): *δ* 7.20 (s, 4H, CH–Ar), 3.63 (s, 4H, N–CH_2_–Ar), 3.22 (m, 4H, N–CH_2_), 3.01 (m, 4H, N–CH_2_), 2.82 (m, 4H, N–CH_2_), 2.61 (m, 4H, N–CH_2_), 2.52 (m, 8H, N–CH_2_), 2.44 (m, 12H, N–CH_2_), 2.14 (m, 4H, N–CH_2_), 2.01 (s, 6H, CH_3_), 1.75 (m, 4H, N–CH_2_), 1.66 (m, 4H, N–CH_2_). ^13^C NMR (CDCl_3_): *δ* 19.51 (N– –CH_2_), 20.45 (N– –CH_2_), 41.12 (N–CH_3_), 44.73 (N–CH_2_), 49.13 (N–CH_2_), 51.02 (N–CH_2_), 54.21 (N–CH_2_), 57.43 (N–CH_2_), 59.31 (N–CH_2_), 63.55 (N–CH_2_–Ar), 122.82 (Ar–CH), 141.22 (Ar–C). Elemental analysis: calculated (%) for C_34_H_62_N_8_·1.35H_2_O: C, 67.25; H, 10.74; N, 18.45. Found: C, 67.18; H, 10.59; N, 18.63. MS (ESI): *m*/*z* = 583.5 ([M + H]^+^). HRMS (ESI): calculated for [M + H]^+^, 583.5170; found 583.5167.

#### Ligand 3

Amounts used: [Ni_2_3]^4+^ (1 g, 0.7 mmol) in 20 mL water/MeCN, sodium cyanide (350 mg, 7 mmol). Yield (465 mg, 90%). ^1^H NMR (CDCl_3_): *δ* 7.20–7.37 (s, 14H, C–H–Ar), 3.61 (s, 8H, N–CH_2_–Ar), 3.15 (m, 4H, N–CH_2_), 2.97 (m, 4H, N–CH_2_), 2.79 (m, 4H, N–CH_2_), 2.57 (m, 4H, N–CH_2_), 2.46 (m, 8H, N–CH_2_), 2.24 (m, 12H, N–CH_2_), 2.04 (m, 4H, N–CH_2_), 1.69 (m, 4H, N–CH_2_), 1.62 (m, 4H, N–CH_2_). ^13^C NMR (CDCl_3_): *δ* 22.53 (N– –CH_2_), 23.47 (N– –CH_2_), 47.12 (N–CH_2_), 49.11 (N–CH_2_), 57.73 (N–CH_2_), 59.28 (N–CH_2_), 66.12 (N–CH_2_–Ar), 67.19 (N–CH_2_–Ar), 118.44 (Ar–CH), 130.07 (Ar–CH), 131.94 (Ar–CH), 133.44 (Ar–CH), 141.22 (Ar–C), 144.18 (Ar–C). Elemental analysis: calculated (%) for C_46_H_70_N_8_·0.8H_2_O: C, 73.71; H, 9.63; N, 14.95. Found: C, 73.70; H, 9.52; N, 14.79. MS (ESI): *m*/*z* = 735.58 ([M + H]^+^).

### General procedure for metal complex formation reaction

The macrocycle ligand was dissolved in degassed anhydrous MeOH. A solution of metal(ii) acetate in anhydrous MeOH was added dropwise to the mixture at 80 °C. The reaction was heated to reflux for 24 h. Solvent was removed *in vacuo* to 1 mL and the complex was purified *via* size exclusion chromatography (Sephadex LH20). Solvent was removed to yield the desired compound.

#### [Cu1]^2+^

Amounts used: ligand 1 (200 mg, 0.6 mmol) in methanol (10 mL), copper acetate monohydrate (132 mg, 0.66 mmol) in methanol (5 mL). Yield a green–blue solid (290 mg, 94%). Elemental analysis: calculated (%) for [C_20_H_34_N_4_Cu](CH_3_COO)_2_·2.6H_2_O·1MeOH: C, 50.80; H, 8.39; N, 9.48. Found (%): C, 50.92; H, 8.47; N, 9.18. MS (ESI): *m*/*z* = 452.22 ([[C_20_H_34_N_4_Cu](CH_3_COO)]^+^). HRMS (ESI): calculated for [C_20_H_34_N_4_Cu]^2+^, 196.6034; found 196.6030. UV-vis (H_2_O): *λ*_max_ = 533 nm.

#### [Zn1]^2+^

Amounts used: ligand 1 (200 mg, 0.6 mmol) in methanol (10 mL), zinc acetate dihydrate (145 mg, 0.66 mmol) in methanol (5 mL). Yield a yellow solid (290 mg, 94%). Elemental analysis: calculated (%) for [C_20_H_34_N_4_Zn](CH_3_COO)_2_·2.2H_2_O·1.2MeOH: C, 51.12; H, 8.38; N, 9.46. Found (%): C, 50.99; H, 8.43; N, 9.52. MS (ESI): *m*/*z* = 453.22 ([[C_20_H_34_N_4_Zn](CH_3_COO)]^+^). HRMS (ESI): calculated for [[C_20_H_34_N_4_Zn](CH_3_COO)]^+^, 453.2202; found 453.2222. UV-vis (H_2_O): *λ*_max_ = 526 nm.

#### [Cu_2_2]^4+^

Amounts used: ligand 2 (200 mg, 0.34 mmol) in methanol (10 mL), copper acetate monohydrate (150 mg, 0.75 mmol) in methanol (5 mL). Yield a purple-blue solid (300 mg, 93%). Elemental analysis: calculated (%) for [C_34_H_62_N_8_Cu_2_](CH_3_COO)_4_·1.2H_2_O·1MeOH: C, 51.65; H, 8.11; N, 11.21. Found (%): C, 51.62; H, 8.17; N, 11.25. HRMS (ESI): calculated for [[C_34_H_62_N_8_Cu_2_](CH_3_COO)_2_]^2+^, 413.1972; found 413.1966. UV-vis (H_2_O): *λ*_max_ = 518 nm.

#### [Zn_2_2]^4+^

Amounts used: ligand 2 (200 mg, 0.34 mmol) in methanol (10 mL), zinc acetate dihydrate (137 mg, 0.75 mmol) in methanol (5 mL). Yield a yellow solid (270 mg, 92%). Elemental analysis: calculated (%) for [C_34_H_62_N_8_Zn_2_](CH_3_COO)_4_·2H_2_O·0.8MeOH: C, 50.82; H, 8.09; N, 11.08. Found (%): C, 50.89; H, 8.03; N, 11.18. MS (ESI): *m*/*z* = 415.20 ([[C_34_H_62_N_8_Zn_2_](CH_3_COO)_2_]^2+^). HRMS (ESI): calculated for [[C_34_H_62_N_8_Zn_2_](CH_3_COO)_2_]^2+^, 415.1956; found 415.1949. UV-vis (H_2_O): *λ*_max_ = 536 nm.

#### [Cu_2_3]^4+^

Amounts used: ligand 3 (100 mg, 0.14 mmol) in methanol (6 mL), copper acetate monohydrate (62 mg, 0.31 mmol) in methanol (2 mL). Yield a purple-blue solid (125 mg, 82%). Elemental analysis: calculated (%) for [C_46_H_70_N_8_Cu_2_](CH_3_COO)_4_·0.8H_2_O·0.7MeOH: C, 57.87; H, 7.67; N, 9.87. Found: C, 57.80; H, 7.65; N, 9.83. MS (ESI): *m*/*z* = 489.23 ([[C_46_H_70_N_8_Cu_2_](CH_3_COO)_2_]^2+^). HRMS (ESI): calculated for [[C_46_H_70_N_8_Cu_2_](CH_3_COO)_2_]^2+^, 489.2281; found 489.2274. UV-vis (H_2_O): *λ*_max_ = 502 nm.

#### [Zn_2_3]^4+^

Amounts used: ligand 2 (100 mg, 0.14 mmol) in methanol (6 mL), zinc acetate dihydrate (68 mg, 0.31 mmol) in methanol (2 mL). Yield a yellow solid (120 mg, 78%). Elemental analysis: calculated (%) for [C_46_H_70_N_8_Zn_2_](CH_3_COO)_4_·0.9H_2_O·0.7MeOH: C, 57.60; H, 7.65; N, 9.82. Found (%): C, 57.58; H, 7.62; N, 9.80. MS (ESI): *m*/*z* = 491.23 ([[C_46_H_70_N_8_Zn_2_](CH_3_COO)_2_]^2+^). HRMS (ESI): calculated for [[C_46_H_70_N_8_Zn_2_](CH_3_COO)_2_]^2+^, 492.2264; found 492.2247. UV-vis (H_2_O): *λ*_max_ = 535 nm.

### Biological evaluation

#### Cell lines and compounds

Peripheral blood mononuclear cells (PBMCs) activated with phytohemagglutinin (PHA; Sigma, Belgium) were used for CXCL12 binding studies. The glioblastoma astrocytoma U87.CD4 cell line transfected with CXCR4 was used for measurement of CXCL12-induced calcium mobilization. The MT-4 cell line was previously described.^43^ The CXCR4 inhibitor AMD3100 (Plerixafor™) was a gift from Dr Gary Bridger affiliated at that time with AnorMED (Langley, Canada). The HIV-1 NL4.3 (CXCR4-using, ×4) strain was obtained through the AIDS Research and Reference Reagent Program (Division of AIDS, NIAID, NIH).

#### HIV infection assay

MT-4 cells were seeded (1 × 10^4^ cells per well) in transparent 96-well plates in DMEM (Dulbecco's Modified Eagle Medium; Life Technologies, Waltham, MA, USA) supplemented with 10% Foetal Bovine Serum (FBS) and 10 mM HEPES. Subsequently, compounds were added and the cell/compound mixture was incubated at 37 °C for 30 min. Thereafter, HIV-1 NL4.3 was added (100 pg p-24 per well) and incubated with the cells for 48 h. The HIV-1 induced cytopathogenic effect (CPE) was evaluated microscopically and cell viability was quantified using a tetrazolium-based colorimetric assay as described before.^43^

#### Chemokine (CXCL12) binding inhibition assay

Human peripheral blood lymphocytes (PBLs) were washed once with assay buffer (Hanks’ balanced salt solution with 20 mM HEPES buffer and 0.2% bovine serum albumin, pH 7.4) and then incubated for 15 min at room temperature with CXCR4 antagonists, at a range of concentrations, diluted in assay buffer. Subsequently, AlexaFluor647-labelled CXCL12 (CXCL12-AF647) (25 ng mL^−1^) was added (30 min, room temperature) to the compound-incubated cells. Thereafter, the cells were washed twice in assay buffer, fixed in paraformaldehyde (1%) in PBS and analysed on the FL4 channel of a FACSCalibur flow cytometer equipped with a 635 nm red diode laser (Becton Dickinson, San Jose, CA, USA). The percentage inhibition of CXCL12-AF647 binding was calculated according to the formula: [1 − ((MFI − MFI_NC_)/(MFI_PC_ − MFI_NC_))] × 100 where MFI is the mean fluorescence intensity of the cells incubated with CXCL12-AF647 in the presence of the inhibitor, MFI_NC_ is the mean fluorescence intensity measured in the negative control (*i.e.*, autofluorescence of unlabeled cells) and MFI_PC_ is the mean fluorescence intensity of the positive control (*i.e.*, cells exposed to CXCL12-AF647 alone).

#### Measurement of intracellular CXCL12-induced calcium mobilization

CXCR4-positive U87 glioblastoma/astrocytoma cells were loaded (45 min, room temperature) with the fluorescent calcium indicator Fluo-3 acetoxymethyl (Molecular Probes) (4 μM) in assay buffer (Hanks’ balanced salt solution with 20 mM HEPES buffer and 0.2% bovine serum albumin, pH 7.4). After thorough washing with assay buffer, cells were pre-incubated (10 min, 37 °C) in the same buffer with the various CXCR4 antagonists at a range of concentrations. The intracellular calcium mobilization in response to CXCL12 addition was then measured by monitoring the fluorescence as a function of time in all the wells simultaneously by using a Fluorometric Imaging Plate Reader (FLIPR; Molecular Devices, Sunnyvale, CA, USA).^40,41^

#### Determination of cellular toxicity

Cytotoxicity measurements in MT-4 cells were based on the viability of the cells that had been exposed to various concentrations of the test compound.^44^ After the cells were allowed to proliferate for 5 days, the number of viable cells was quantified by a tetrazolium-based colorimetric method, as originally described by Pauwels *et al.*^45^

## Conclusions

Some conclusions can be drawn about the potential for *trans*-IV configuration cyclam complexes as high affinity CXCR4 binding agents, for example, in comparison with the structurally similar *trans*-II configuration. Whilst nickel(ii), copper(ii) and zinc(ii) complexes as *trans*-II structures are all highly active (in the order zinc > nickel > copper),^21,25,27^ transition metal ion selection has a significant effect on the binding of *trans*-IV complexes. Neither of the nickel(ii) *trans*-IV compounds developed in this study demonstrated low nanomolar affinity for the CXCR4 receptor. Of the three fixed configurations studied to date (*trans*-II, *cis*-V and *trans*-IV), *trans*-IV is the most variable in CXCR4 binding properties across the range of transition metals tested, with copper(ii) and zinc(ii) complexes showing superiority to AMD3100 in some assays. Targeting CXCL12 binding, and subsequent CXCR4 receptor activation, is the key step in developing potent CXCR4 inhibition agents for cancer therapy. A further useful feature is the facile functionalization of the *trans*-IV structure, using the methodologies presented in this study. Variation in substituents can have a major impact on CXCR4 binding, as observed for the copper(ii) complex. This is consistent with the sensitivity of the binding affinity to the coordination environment for copper(ii) due to its coordination properties.

Overall, the high affinity and inhibitory action of zinc(ii) *trans*-IV structures (compared to AMD3100) coupled with the versatility in substitution to tune the molecular properties, opens the door to a new sub-class of compounds that may exhibit increased drug efficacy and improved properties as potential CXCR4-targeted anti-cancer agents.

## Author contributions

Conceptualisation: S. O. A., G. Mc., D. S., S. J. A.; Methodology: G. Mc., B. P. B., T. J. H., D. S., S. J. A.; Validation: B. P. B., I. R.; Investigation: S. O. J., G. Mc., A. K., T. D'H, T. V. L., A. N. W., I. R.; Writing – original draft: S. O. A., G. Mc., T. D'H., T. V. L., I. R., D. S., B. P. B., S. J. A.; Writing – review & editing: A. K., I. R., T. J. H., D. S., B. P. B., S. J. A.; Supervision: T. J. H., D. S., S. J. A.; Funding acquisition: D. S., S. J. A.

## Conflicts of interest

There are no conflicts to declare.

## Supplementary Material
